# Evaluation of plant elicitation with methyl-jasmonate, salicylic acid and benzo (1,2,3)-thiadiazole-7-carbothioic acid-S-methyl ester for the sustainable management of the pine wilt disease

**DOI:** 10.1093/treephys/tpac088

**Published:** 2022-07-22

**Authors:** Adrián López-Villamor, Marta Nunes da Silva, Marta W Vasconcelos

**Affiliations:** Universidade Católica Portuguesa, CBQF – Centro de Biotecnologia e Química Fina-Laboratório Associado, Escola Superior de Biotecnologia, Rua de Diogo Botelho 1327, 4169-005 Porto, Portugal; Misión Biológica de Galicia (CSIC), Grupo de Genética y Ecología Forestal, Apdo. 28, 36080 Pontevedra, Spain; Universidade Católica Portuguesa, CBQF – Centro de Biotecnologia e Química Fina-Laboratório Associado, Escola Superior de Biotecnologia, Rua de Diogo Botelho 1327, 4169-005 Porto, Portugal; Universidade Católica Portuguesa, CBQF – Centro de Biotecnologia e Química Fina-Laboratório Associado, Escola Superior de Biotecnologia, Rua de Diogo Botelho 1327, 4169-005 Porto, Portugal

**Keywords:** antioxidant system, bacterial population, *Bursaphelenchus xylophilus*, pine wilt nematode, *Pinus pinaster*, tolerance

## Abstract

Treatment with plant elicitors can be a promising method to induce *Pinus pinaster* tolerance against the pinewood nematode (PWN), *Bursaphelenchus xylophilus*, by promoting plant antioxidant system, micronutrient accumulation and by modulating plant-associated bacterial populations. To test this hypothesis, plants were sprayed with methyl jasmonate (MeJA), salicylic acid (SA) or benzo (1,2,3)-thiadiazole-7-carbothioic acid-S-methyl ester (BTH), and evaluated until 35 days after-inoculation (dai) for: i) extent of foliar symptoms; ii) nematode density inside stem tissues; iii) proxies for oxidative damage and antioxidant activity, iv) micronutrient concentration and v) bacterial diversity. Compared with non-elicited plants, plant elicitation, particularly with BTH, significantly decreased nematodes density inside stem tissues (by 0.63-fold). Concordantly, without elicitation plant mortality reached 12.5% while no mortality was observed in elicited plants. BTH-elicited plants had significantly higher concentrations of anthocyanins and carotenoids at the end of the assay than SA-elicited and MeJA-elicited plants, which possibly contributed to the lower PWN colonization and degree of foliar symptoms observed. Accordingly, MeJA and SA led to increased lipid peroxidation at 28 dai (by 2.64- and 2.52-fold, respectively) in comparison with BTH (by 1.10-fold), corroborating its higher potential in increasing plant antioxidative response during infection. Moreover, carotenoids showed a negative correlation with nematode migration, whereas polyphenols showed a positive correlation. Elicitors also induced changes in the bacterial community of infected *P. pinaster* plants, increasing the diversity of specific populations. Finally, elicitors induced significant changes in micronutrients accumulation in plant tissues, namely a decrease in the concentration of B, Mn and Ni in plants treated with BTH compared to those treated with the other elicitors. Altogether, results suggest that elicitation with MeJA, SA and, particularly, BTH, increases tolerance against *B. xylophilus* by promoting plant antioxidant system, changing the accumulation of essential micronutrients and modulating plant-associated bacterial diversity.

## Introduction

*Pinus* spp. is the most used genus in industrial forest plantations worldwide (Mbabazi 2011). The maritime pine (*Pinus pinaster)* is particularly relevant for the timber industry and can be found in several Western European countries, such as Portugal, Spain, France and in some Northern African countries (Chupin et al. 2015). In spite of the economic and social importance of *P. pinaster*, in recent years there has been a marked reduction of production with significant losses in area and volume due to forest fires, but also due to the propagation of the pinewood nematode (PWN), *Bursaphelenchus xylophilus* (Abad et al. 2016). The PWN constitutes one of the most serious worldwide pathogens, affecting native species of *Pinus* spp. from Japan, China, Korea and Taiwan, and also reaching Europe (Portugal and Spain) (Vicente et al. 2012); it is considered a major threat to forestry ecosystems and a quarantine pest by the European and Mediterranean Plant Protection Organization (EPPO) (EPPO 2014).

Due to the serious deterioration of *Pinus* spp. forests worldwide caused by the PWN, many efforts have been made to contain the progression of this disease. The early control methods against the pine wilt disease (PWD) were based on aerial and ground applications of insecticides to eliminate the insect vectors of the PWN, and on the injection of pine plants with nematicides (Lee et al. 2003, Kurinobu 2008, Vicente et al. 2012). Recently, there have been growing concerns regarding the use of nematicides and pesticides, owing to their injurious effects on the environment and human health, as well as to undesirable effects on non-target organisms (Park et al. 2007, Vicente et al. 2012).

Breeding programs to search for and implement tolerance against the PWN (Kurinobu 2008, Nose and Shiraishi 2008, Carrasquinho et al. 2018, Menéndez-Gutiérrez et al. 2018), which are expensive, time consuming, and can only be applied to new plantations; the use of ectomycorrhizal fungi (Chu et al. 2019, Nakashima et al. 2016); the induction of systemic acquired resistance (SAR) (Salas-Marina et al. 2011, Mannaa et al. 2020, Park et al. 2020, Jeon et al. 2022), which is activated for biotrophic and hemi-biotrophic pathogens and mediated by salicylic acid (SA) (Van Loon 2007, Khan et al. 2015, Klessig et al. 2018, Tripathi et al. 2019) and the induced systemic resistance (ISR), which is associated with the perception of necrotrophic pathogens and herbivorous insects and mediated by jasmonic acid (JA) and ethylene (ET) (Bari and Jones 2009, Kolosova and Bohlmann 2012) may provide viable alternatives to currently used control agents. However, the PWD is a complex disease that affects multiple systemic aspects of the pathosystem, hampering the success of disease control strategies.

When the nematode enters the plant host, it moves and reproduces within the resin canals, causing a general oxidative damage that results in visible necrosis in the leaves (Kuroda 2008, Yamada 2008). To counteract the detrimental effects of these oxidative molecules, plants activate several antioxidant enzymes, such as superoxide dismutase, peroxidases and catalase. Therefore, the induction of SAR and ISR through the application of elicitors could improve the tolerance of *P. pinaster* against oxidative damage caused by PWN. However, if excessive oxidative stress occurs, plant cells may accumulate malondialdehyde (MDA), a secondary product of cell wall lipid peroxidation (Heath and Packer 1968, Nunes da Silva et al. 2015), soluble phenolic compounds associated with the browning of the leaf tissues injured by the PWN (Treutter 2006) and anthocyanin and carotenoids, photosynthetic pigments that act in defense against oxidative stress (Elkhouni et al. 2018).

Furthermore, it is well known that non-metallic micronutrients, such as B, are essential constituent of cell walls (Blevins and Lukaszewski 1998), and proper cell integrity is key for PWN tolerance. It has been demonstrated that the application of elicitors such as JA and SA can help plants to tolerate toxic levels of different micronutrients (B, Cu, Mn, Ni and Zn) (El-Tayeb et al. 2006, Sheng et al. 2015, Yavas and Unay 2016, Metwally et al. 2018, Mir et al. 2018, Zaid et al. 2019, Ali and Baek 2020, Dai et al. 2020). Micronutrients also play an important role in plant tolerance to biotic and abiotic stress (particularly in resistance to pests and diseases) (Kirkby and Römheld 2004). For example, they function as co-factors of several metalloproteins (Fe, Mn, Cu and Ni), they activate enzymatic reactions (Mn and Zn) and are generally involved in stress tolerance (Mn and Zn) (Asher et al. 1991, Bergmann and Caesar 1994, Mengel and Kirkby 2001, Epstein and Bloom 2005, Marschner 2011). Elicitors can affect the mineral composition (Cu, Fe, Mn and Zn) of plant tissues in response to biotic and abiotic stresses (Ghassemi-Golezani and Farhangi-Abriz 2018, Débia et al. 2020). However, the impact of MeJA, SA and BTH elicitation in micronutrient accumulation in pine plants, and the potential repercussion on plant susceptibility to the PWN has not been studied yet.

On the other hand, plant-associated bacterial communities also play an important role in the absorption of certain nutrients (Zhang et al. 2018, Wang et al. 2020), plant growth-promotion (Gu et al. 2020) and in defense against pathogens (Burketova et al. 2015, Doornbos et al. 2012). Although the bacterial communities associated with *P. pinaster* and PWN have been under study in the past decade (Proença et al. 2010, 2017, Roriz et al. 2011, Vicente et al. 2011), the effect of MeJA, SA and BTH on the modulation of bacterial diversity has never been evaluated and we hypothesize they may have the potential to induce pine defenses against the PWN via a systemic modulation of multiple plant defense responses.

The aim of this study was to assess the effectiveness of MeJA, SA and BTH as tools to induce plant tolerance against the PWN through a comprehensive evaluation of: i) nematode progression in plant tissues; ii) foliar symptoms and photosynthetic pigments; iii) proxies for plant defensive capability and oxidative damage (carotenoids, anthocyanins, total polyphenolics, flavonoids and lipid peroxidation), iv) plant-associated bacterial populations and v) micronutrient profile (B, Cu, Fe, Mn, Ni and Zn).

## Materials and methods

### Plant material and experimental design

Seeds of *P. pinaster* from French-Landes provenance region were planted in 2 L containers filled with peat and perlite (3:1, v:v). A total of 150 2-year-old plants were grown in the greenhouses of Misión Biológica de Galicia-CSIC (MBG-CSIC, Pontevedra, Spain; 42.4054° N, 8.6426° W). The average height and diameter of the plants used were 124 ± 14 and 0.89 ± 0.05 cm, respectively. These plants were transferred to Centro de Biotecnologia e Química Fina-Universidade Católica Portuguesa (CBQF-UCP, Porto, Portugal; 41.1539° N, −8.6733° W), where the experiments took place. These were carried out from 9 April to 14 May 2019, keeping the plants under natural environmental conditions.

### Plant elicitation

Seven days before infection with *B. xylophilus*, the selected elicitors were applied in separate places to avoid cross-contamination between treatments: 33 plants were sprayed with 25 mM of 95% MeJA (Sigma-Aldrich, Missouri, USA) in deionized water with 2.5% ethanol (v/v) (Zas et al. 2019); 33 plants were sprayed with 1 mM SA (Sigma-Aldrich, Missouri, USA) in deionized water with 1% ethanol (v/v) (Vimala and Suriachandraselvan 2009, Tierranegra-García et al. 2011), and 33 plants were sprayed with a 1 mM suspension of BTH (Sigma-Aldrich, Missouri, USA) in deionized water (Conejero et al. 2012). A separate group of 51 plants were used as non-elicited control plants of which 33 were sprayed with deionized water. All solutions were sprayed over the aboveground part of each plant to run off (20 ± 1.5 mL per plant), being an adaptation of the method used by Zas et al. (2019).

### Plant inoculation

Seven days after plant elicitation, a virulent strain of *B. xylophilus* (strain 17AS) was used for inoculation. The nematodes were maintained in mycoboxes with *Botrytis cinerea* (Pers) mycelia growing in barley seeds at 25°C for 14 days. Nematodes were extracted from the culturing medium using the Baermann funnel technique (Baermann 1917) for 24 h at 25°C and their density was adjusted so that a solution with 2000 nematodes in 750 μl of sterilized water was obtained. Inoculation was performed as described by Futai (2003). Briefly, at ~20 cm from the top of each plant, leaves were removed from a 3 cm portion of the stem, and transversal cuts were made using a sterile blade. A piece of absorbent paper was placed around the wound, the nematode suspension was pipetted, and parafilm was used to seal the inoculation site. Non-elicited plants and plants previously elicited with MeJA, SA or BTH (33 plants for each treatment) were inoculated with PWN, resulting in four inoculated treatments: inoculated non-elicited controls (iCTR), inoculated MeJA-elicited plants (iMeJA), inoculated SA-elicited plants (iSA) and inoculated BTH-elicited plants (iBTH). A group of 18 plants served as non-inoculated, non-elicited control plants (niCTR).

### Scoring of foliar symptoms and sampling

Eight plants from each group were used to evaluate disease progression through visual analysis of leaf foliar symptoms at five different time-points: 7, 14, 21, 28 and 35 days after inoculation (dai). The degree of wilting and defoliation was visually assessed on a 0–4 scale: 0 = 0–10% symptomatic leaf tissue; 1 = 11–33%; 2 = 34–66%; 3 ≥67% and 4 = total leaf wilting or defoliation (Sánchez et al. 2005).

Plant sampling was performed at the same time-points (7, 14, 21, 28 and 35 dai). The leaves of five plants randomly selected from each treatment were separated from the stems, ground to a fine powder with liquid nitrogen, and used for chlorophyll, lipid peroxidation, total soluble phenols and total flavonoids content and mineral quantification, whereas stems were used for whole-stem nematode quantification and microbiological analysis.

### Nematode quantification

The leaves of plants used for nematode quantification (*n* = 5) were removed, and stems were cut into small portions (*~*0.5 cm). Nematode were extracted from stems using the Baermann funnel technic for 24 at 25°C, and quantified using a nematode counting dish under a transmitted light stereo microscope, as described by Nunes da Silva et al. (2015).

### Primary and secondary metabolites

For total chlorophyll and carotenoids quantification, the Sims and Gamon (2002) method was used. In brief, 0.1 g of leaf tissue was mixed with 10 ml of cold acetone/Tris buffer solution at 1 M (80:20, v:v, pH = 7.8) and incubated at 4°C for 24–72 hours, after which samples were centrifuged at 13,000 rpm for 5 min. Using the NanoPhotometer™ UV/VIS spectrometer (Implen GmbH, Germany) absorbances were recorded at 470, 537, 647 and 663 nm, and the concentration of pigments was calculated as follows, taking into consideration the sample fresh weight:

Anthocyanin = 0.08173*A*_537−_0.00697*A*_647−_0.002228*A*_663_

Chl_a_ = 0.01373*A*_663_–0.000897*A*_537_–0.003046*A*_647_

Chl_b_ = 0.02405*A*_647−_0.004305*A*_537_–0.005507*A*_663_

Carotenoids = (A_470_—(17.1 x (Chl_a_ + Chl_b_)—9.479 x Anthocyanin)/119.2

For the quantification of soluble phenols and flavonoids, 50 mg of lyophilized leaf tissue was extracted with 1.5 ml of 80% aqueous methanol (v:v) in an ultrasound bath for 20 min. The extract was recovered after centrifugation at 15,000 g for 15 min.

Total soluble phenolics were determined according to the Folin-Denis’ method (Marinova et al. 2005). Firstly, 4.5 ml of ultrapure water and 500 μl of Folin-Denis’ reagent was added to 100 μl of methanolic extract. The mixture was stirred vigorously mixed and the reaction allowed to occur for 5 min, after which 5 ml of sodium carbonate at 7% (w:v) was added. After incubation at room temperature in the dark for 1 h, 2 ml of ultrapure water was added to each sample. The absorbances were recorded at 750 nm using a NanoPhotometer™ UV/VIS spectrometer (Implen GmbH, Germany) and the concentration of total soluble phenolics determined using a gallic acid calibration curve.

For flavonoids determination, the aluminum chloride method (Zhishen et al. 1999) was used. Namely, 2 ml of ultrapure water and 150 μl of NaNO_2_ at 5% were added to 100 μl of methanolic extract. The mixture was incubated for 5 min at room temperature. Afterwards, 150 μl of AlCl_3_ at 10%, 1 ml of 1 M NaOH and 1.2 ml of ultrapure water were added. The absorbances were recorded at 510 nm using a NanoPhotometer™ UV/VIS spectrometer (Implen GmbH, Germany) and flavonoids concentration was determined using a catechin calibration curve.

### Quantification of lipid peroxidation

Determination of lipid peroxidation was performed through malondialdehyde (MDA) quantification, following a modified version of the protocol described by Li (2000). In brief, 10 ml of 0.5% thiobarbituric acid in 20% trichloroacetic acid were added to 0.1 g of leaf sample. Each sample was homogenized through vigorous agitation for 30 s and incubated in a water bath at 100°C for 30 min. After the incubation period, the reaction was terminated by transferring the samples into ice. Samples were centrifuged for 10 min at 5000 rpm and the supernatant was filtrated. The absorbance was measured at 450, 532 and 600 nm and MDA was quantified through the equation:

MDA (μmol. L^−1^) = 6.45 x (*A*_532_ – *A*_600_) – 0.56 x *A*_450_

### Mineral determination by ICP-OES

For mineral determination, three plants were randomly selected from each treatment at 28 dai. Leaf samples (*~*0.2 g) were mixed with 5 ml of 65% HNO_3_ in a Teflon reaction vessel and heated in a SpeedwaveTM MWS-3þ (Berghof, Germany) microwave system. The digestion procedure was conducted in five steps, consisting of different temperature and time sets: 130°C/10 min, 160°C/15 min, 170°C/12 min, 100°C/7 min and 100°C/3 min (Santos et al. 2015). The resulting clear solutions of the digestion procedure were then adjusted to 50 mL with ultrapure water for further analysis. Mineral determination was performed using the inductively coupled plasma optical emission spectrometer (ICP-OES) Optima 7000 DV (PerkinElmer, USA) with radial configuration. For each sample, two technical replicates were prepared.

### Extraction and isolation of plant-associated bacterial populations

Analysis of plant bacterial population was carried out in all plants at the end of the assay (35 days after infection). The stems of three plants randomly selected from each treatment were separated into small portions (*~*2 cm), which were sterilized by submerging in 75% ethanol for 15 s, and the excess ethanol was removed by washing in deionized water (Xie and Zhao 2008). The extremities of each stem segment were removed in aseptic conditions, and each segment was cut horizontally and placed in nutrient agar (NA) medium with the vascular tissue facing down. After incubation at 26°C for 3 days, morphologically distinct bacterial colonies were identified and isolated until pure cultures were obtained. For each treatment, three plants were used, and for each plant six stem portions and two replicates were analyzed.

### Molecular identification of the bacterial populations

For the molecular identification of the bacterial cultures obtained as described before, the total genomic DNA of each bacterial isolate was extracted using the heat-shock method as performed by Calheiros et al. (2010). Colonies were added to 200 μl of sterile ultra-pure water, homogenized through vigorous stirring and incubated at 95°C for 10 min. Samples were then transferred into ice for 5 min, vortexed and centrifuged at 15,000 rpm for 5 min in a microcentrifuge (Heraeus Pico 17, Thermo Scientific, USA). The concentration and integrity of the extracted DNA was evaluated spectrophotometrically using a NanoPhotometer™ UV/VIS spectrometer (Implen GmbH, Germany).

16S rRNA genes were amplified by PCR using 12.5 μl of NZYTaq II 2x Green Master Mix (NZYTech, Portugal) with 0.5 μM of primers 27F (5’-GAGTTTGATCCTGGCTCA-3′) and 1493R (5’-TACCTTGTTACGACTT-3′), and 5 μl of bacterial DNA in a total volume of 25 μl. The PCR reactions were performed on a thermocycler DOPPIO (VWR, USA) using the parameters: 1 cycle of initial denaturation at 95°C for 120 s, 25 cycles of denaturation at 95°C for 30 s, annealing at 54°C for 30 s and extension at 72°C for 1 min and finally one cycle of a final extension at 72°C for 5 min. The final product was analyzed by electrophoreses in a 1% agarose gel in Tris-EDTA (TAE) buffer with DNA stains Gel Red™ (Biotium, Inc., USA) for 45 min at 120 V and 400 mA. PCR products of all 47 bacterial isolates were sequenced by STAB VIDA, Lda. (Lisbon, Portugal) and identified using the Basic Local Alignment Search Tool (blastN, National Center for Biotechnology Information, USA).

**Figure 1.**
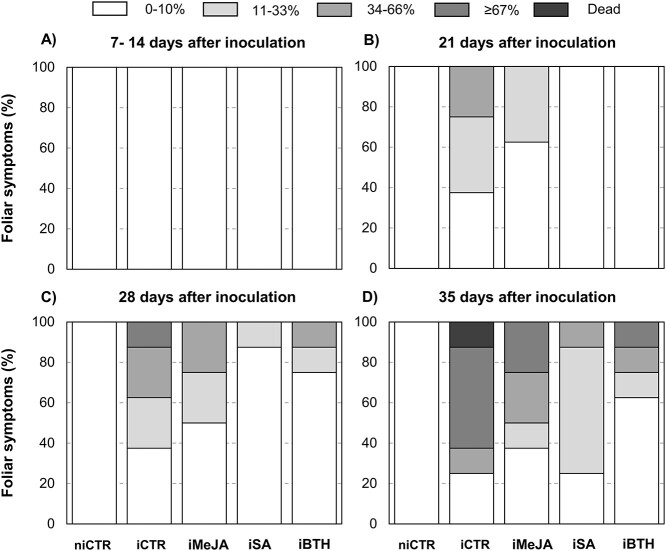
Foliar symptoms (%) at (**A**) 7–14 days, (**B**) 21 days, (**C**) 28 days and (**D**) 35 dai in non-infected non-treated control plants (niCTR), infected non-treated control trees (iCTR), infected trees treated with methyl-jasmonate (iMeJA), salicylic acid (iSA) or benzo (1,2,3)-thiadiazole-7-carbothioic acid-S-methyl ester (iBTH).

### Statistical analysis

Results were analyzed using GraphPad Prism v.8 (GraphPad Software, USA). Effect of time-point (Tp) and plant treatments (T) and their interaction (T x Tp) on the number of nematodes, anthocyanin, carotenoids, chlorophyll-A and chlorophyll-B, lipid peroxidation, total soluble phenolics and flavonoids and micronutrient concentration in leaf tissues were analyzed considering T, Tp and their interaction as fixed factors. The significant differences between elicitation treatments were determined using Missed-effects model (REML), which uses the restricted likelihood method and a probability value *P* < 0.05 as the threshold level of significance. The correlation between the different variables measured at 28 dai were determined using Pearson’s correlation matrix.

## Results

### Disease symptoms and nematode population

The results of the foliar damage are shown in Figure 1. Non-infected non-treated control plants (niCTR) did not present foliar symptoms. Foliar damage was only observed in infected plants at 21 days after infection (dai), where 62.5% of the non-treated infected non-treated control plants (iCTR) and 37.5% of infected MeJA-elicited plants (iMeJA) presented varying degrees of foliar damage. Contrastingly, at this time-point, infected SA-elicited (iSA) and BTH-elicited (iBTH) plants did not present any foliar damage. At 28 dai, 72.5% of iCTR plants presented leaf damage, of which 12.5% were in stage 3 (≥67%). iMeJA presented 25% of foliar damage at 28 dai, while iSA and iBTH presented 12.5 and 25% of foliar damage, respectively. At the end of the assay (35 dai), 12.5% of iCTR plants had died, 72.5% presented foliar damage between the stages 3 and 2 (34–66%) and only 25% did not present any foliar damage. Contrastingly, with elicitation no plant mortality was observed, and iMeJA and iBTH resulted in only 72.5% and 37.5% of plants with foliar damage. In iSA, 75% of plants had disease symptoms that did not progress beyond stage 2. The percentage of leaf damage was highly correlated with nematode numbers inside stem tissues (Table 1).

**Table 1:** Pearson’s correlation matrix between the different variables measured at 28 dai: % FDamage (percentage of foliar damage), Nnemat (number of nematodes), Antho (anthocyanins), Carot (carotenoids), phenols (total phenols), LPerox (lipid peroxidation) and micronutrients (B, cu, Fe, Mn, Ni and Zn). Correlation indices indicated by color gradients.

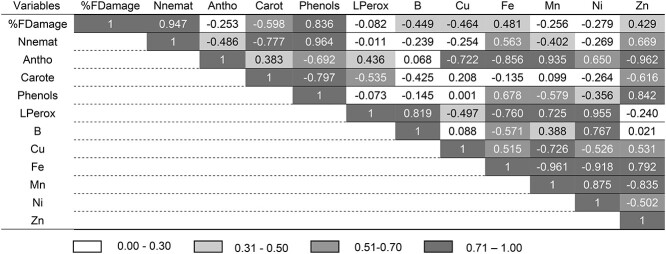

**Figure 2.**
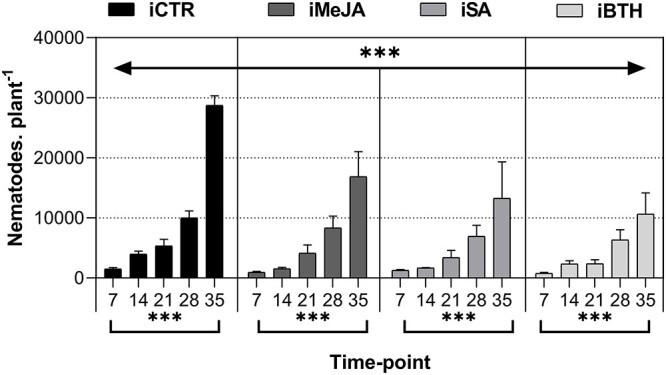
Number of nematodes (Nematodes.Plant^−1^) in infected non-treated control plants (iCTR), infected plants treated with methyl-jasmonate (iMeJA), salicylic acid (iSA) or benzo (1,2,3)-thiadiazole-7-carbothioic acid-S-methyl ester (iBTH) (7, 14, 21, 28 and 35 dai). Values represent the mean of four biological replicates ± standard error of the mean. Significance levels of treatments and time-point for number of nematodes: ^*^^*^^*^, *P* < 0.001; ^*^^*^, *P* < 0.01; ^*^, *P* < 0.05; ns, not significant.

In inoculated plants without elicitor application, nematodes significantly increased from 1528 ± 236 (at 7 dai) to 28,760 ± 2043 (at 35 dai), i.e., 18.82-fold (Figure 2, Table 2). In contrast, in elicited plants, the number of nematodes was significantly lower in all treatments. At 35 dai the number of nematodes inside stem tissues of iMeJA (16,880 ± 4,173), iSA (13,276 ± 6,086) and iBTH (10,642 ± 3,541) were significantly lower than in iCTR (by 1.7-, 2.2- and 2.7-fold, respectively).

**Table 2:** Effect of time-point (Tp, 7, 14, 21, 28 and 35 dai) and plant treatments (T, infected non-treated control plants (iCTR), infected plants treated with methyl-jasmonate (iMeJA), salicylic acid (iSA) or benzo (1,2,3)-thiadiazole-7-carbothioic acid-S-methyl ester (iBTH)) and their interaction (T x Tp) on the number of nematodes, anthocyanin, carotenoids, chlorophyll-a and chlorophyll-B, lipid peroxidation, total soluble phenolics and flavonoids. Significant *P* values (< 0.05) are indicated in bold.

Response variable	Factor	*F* ratio	*P* value
Number of nematodes (nematodes.plant^−1^)	Treatment (T)	8.58	**<0.0001**
	Time-point (Tp)	48.22	**<0.0001**
	T × Tp	3.04	**0.0015**
Anthocyanin (μmol.g^−1^ leaf)	Treatment (T)	8.81	**<0.0001**
	Time-point (Tp)	10.66	**<0.0001**
	T × Tp	3.14	**0.0011**
Carotenoids (μmol.g^−1^ leaf)	Treatment (T)	6.04	**0.0060**
	Time-point (Tp)	7.10	**0.0002**
	T × Tp	3.574	**0.0004**
Chlorophyll-A (μmol.g^−1^ leaf)	Treatment (T)	1.02	0.4084
	Time-point (Tp)	1.15	0.3373
	T × Tp	1.82	0.0642
Chlorophyll-B (μmol.g^−1^ leaf)	Treatment (T)	3.06	0.058
	Time-point (Tp)	4.76	**0.0046**
	T × Tp	3.96	**0.0001**
Total soluble phenolics (mg.g^−1^ leaf)	Treatment (T)	8.75	**<0.0001**
	Time-point (Tp)	5.90	**0.0023**
	T × Tp	10.33	**<0.0001**
Total flavonoid content (mg.g^−1^ leaf)	Treatment (T)	1.88	0.1401
	Time-point (Tp)	2.10	0.1276
	T × Tp	3.67	**0.0002**
Malondialdehyde (μmol.g^−1^ leaf)	Treatment (T)	5.97	**0.0063**
	Time-point (Tp)	21.10	**<0.0001**
	T × Tp	3.41	**0.0007**

### Primary and secondary metabolites

**Figure 3.**
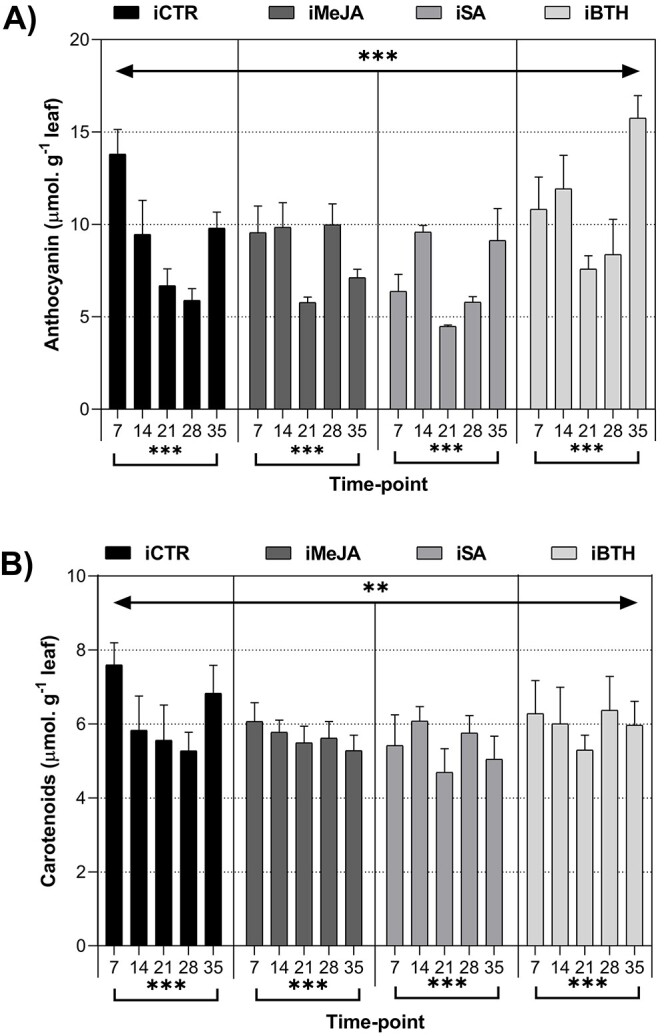
(**A**) Anthocyanin (μmol.G ^−1^ leaf) and (**B**) carotenoids (μmol.G ^−1^ leaf) in infected non-treated control plants (iCTR), infected plants treated with methyl-jasmonate (iMeJA), salicylic acid (iSA) or benzo (1,2,3)-thiadiazole-7-carbothioic acid-S-methyl ester (iBTH) (7, 14, 21, 28 and 35 dai). Values represent the mean of four biological replicates ± standard error of the mean. Significance levels of treatments and time-point for anthocyanin and carotenoids: ^*^^*^^*^, *P* < 0.001; ^*^^*^, *P* < 0.01; ^*^, *P* < 0.05; ns, not significant.

In general, both treatments and time-points significantly affected anthocyanin and carotenoid concentrations, with nematode density inside plant tissues having a negative correlation with carotenoids concentration (Figure 3, Table 1). Anthocyanin (Figure 3A) and carotenoids (Figure 3B) accumulation in iCTR plants showed a progressive and significant decrease from 7 to 28 dai (by 0.43- and 0.69-fold, respectively), slightly increasing at 35 dai (reaching 9.80 ± 1.95 and 6.83 ± 0.78 μmol. g^−1^ leaf). In elicited plants, the concentration of anthocyanins gradually decreased until 21 dai, slightly increasing thereafter (Figure 3A). At the end of the experimental period, iBTH presented the highest anthocyanin concentrations (15.76 ± 2.71 μmol. g^−1^ leaf). Regarding the concentrations of carotenoids, iMeJA showed a slight decrease until the end of the experimental period (by 0.87-fold), while iSA and iBTH showed a decrease at 21 (by 0.87- and 0.84-fold, respectively) and 35 dai (by 0.93- and 0.95-fold, respectively).

Contrastingly to what was observed in anthocyanins and carotenoids, total chlorophylls were not significantly affected by PWN inoculation nor by plant elicitation (Table 2, Figure 1S available as Supplementary data at *Tree Physiology* Online).

**Figure 4.**
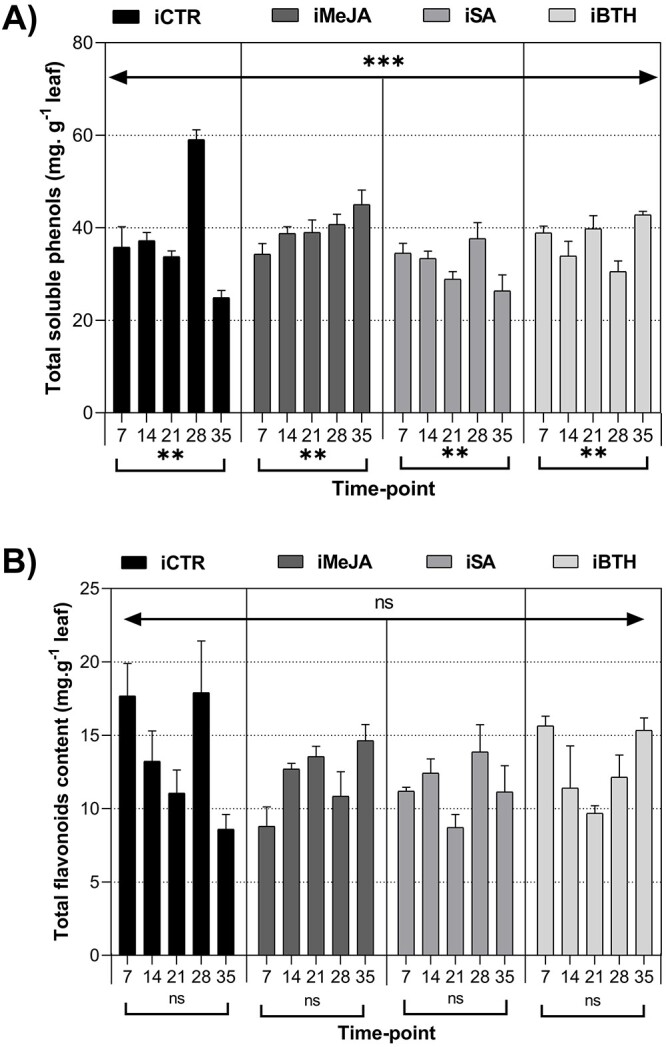
(**A**) Total soluble phenols (mg.Mg^−1^ leaf) and (**B**) total flavonoids content (mg.g^–1^ leaf) in infected non-treated control plants (iCTR), infected plants treated with methyl-jasmonate (iMeJA), salicylic acid (iSA) or benzo (1,2,3)-thiadiazole-7-carbothioic acid-S-methyl ester (iBTH) (7, 14, 21, 28 and 35 dai). Values represent the mean of four biological replicates ± standard error of the mean. Significance levels of treatments and time-point for phenols and flavonoids: ^*^^*^^*^, *P* < 0.001; ^*^^*^, *P* < 0.01; ^*^, *P* < 0.05; ns, not significant.

Treatment and time-point significantly affected total soluble phenols, but not flavonoids content (Table 2). Moreover, a positive correlation was observed between soluble phenols and foliar damage and nematode density, whereas carotenoids were negatively correlated to soluble phenols (Table 1). iCTR and iSA showed a gradual decrease in phenols accumulation along time (by 0.70- and 0.76-fold, respectively), but a significant increase at 28 dai (by 1.65- and 1.09-fold) (Figure 4A). Contrastingly, iMeJA and iBTH showed an increasing trend in soluble phenols, with the highest concentration being recorded at 35 dai (by 1.31- and 1.10-fold, respectively (Figure 4A).

### Lipid peroxidation

**Figure 5.**
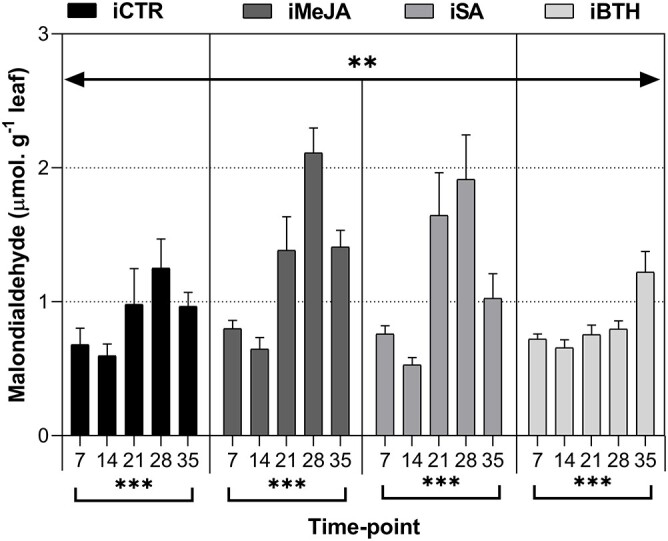
Malondialdehyde (μmol.G ^−1^ leaf) in infected non-treated control plants (iCTR), infected plants treated with methyl-jasmonate (iMeJA), salicylic acid (iSA) or benzo (1,2,3)-thiadiazole-7-carbothioic acid-S-methyl ester (iBTH) (7, 14, 21, 28 and 35 dai). Values represent the mean of four biological replicates ± standard error of the mean. Significance levels of treatments and time-point for malondialdehyde: ^*^^*^^*^, *P* < 0.001; ^*^^*^, *P* < 0.01; ^*^, *P* < 0.05; ns, not significant.

In general, infected plants (both with and without elicitor treatment) displayed a progressive and significant increase in MDA levels until 28 dai, slightly decreasing at 35 dai, except for plants treated with BTH (Figure 5). From 1 to 35 dai, MDA significantly increased in all treatments (from 1.33-fold in iCTR to 1.76-fold in iMeJA).

### Mineral composition

**Figure 6.**
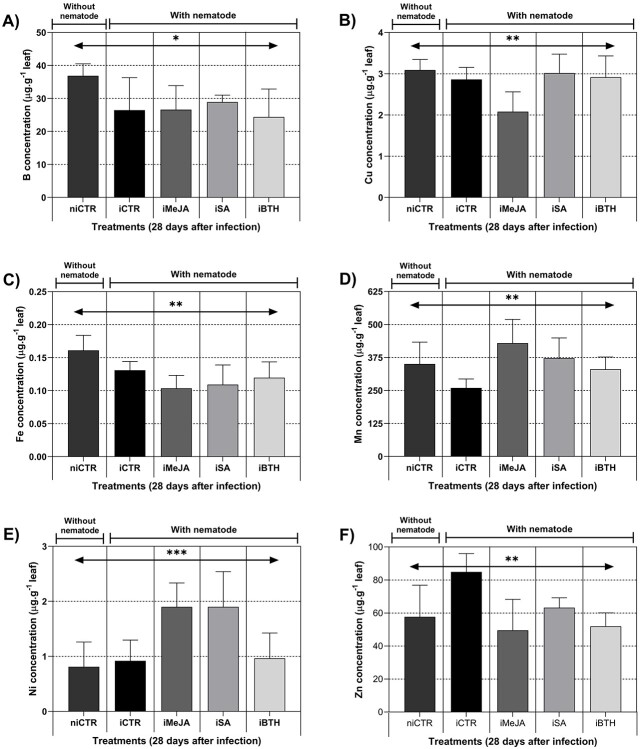
Micronutrients: (**A**) boron (B), (**B**) copper (Cu), (**C**) iron (Fe), (**D**) manganese (Mn), (**E**) nickel (Ni) and (**F**) zinc (Zn) in non-infected non-treated control plants (niCTR), infected non-treated control plants (iCTR), infected plants treated with methyl-jasmonate (iMeJA), salicylic acid (iSA) or benzo (1,2,3)-thiadiazole-7-carbothioic acid-S-methyl ester (iBTH) at 28 days after infection. Values represent the mean of four biological replicates ± standard error of the mean. Significance levels of treatments and time-point for B, Cu, Fe, Mn, Ni and Zn: ^*^^*^^*^, *P* < 0.001; ^*^^*^, *P* < 0.01; ^*^, *P* < 0.05; ns, not significant.

Plant treatments significantly affected the concentration of all micronutrients analyzed (B, Cu, Fe, Mn, Ni and Zn) (Table 3). The highest B concentration was found in niCTR (36.9 ± 3.67 μg.g^−1^ leaf), whereas iBTH had the lowest (24.38 ± 8.48 μg.g^−1^ leaf) (Figure 6A). The average concentrations of Cu were similar between niCTR, iCTR, iSA and iBTH (around 3 ± 0.43 μg.g^−1^ leaf), while iMeJA presented the lowest average concentration (2.08 ± 0.48 μg.g^−1^ leaf), Fe concentration was higher in non-infected control plants (niCTR; 0.16 ± 0.02 μg.g^−1^ leaf), and lowest in iMeJA (0.10 ± 0.02 μg.g^−1^ leaf; Figure 6C). Contrastingly, iMeJA showed the highest Mn concentrations (442.22 ± 90.15 μg.g^−1^ leaf), while iCTR had the lowest (259.41 ± 34.33 μg.g^−1^ leaf; Figure 6D). iMeJA and iSA presented the highest Ni concentrations (1.90 ± 0.45 and 1.90 ± 0.64 μg.g^−1^ leaf, respectively) while niCTR had the lowest concentration (0.81 ± 0.45 μg.g^−1^ leaf; Figure 6E). Finally, iCTR) was the one with the highest Zn concentration (84.88 ± 11.17 μg.g^−1^ leaf), while iBTH and iMeJA had the lowest (51.91 ± 8.20 and 49.54 ± 18.72 μg.g^−1^ leaf, respectively; Figure 6F).

**Table 3:** Effect of plant treatments at 28 dai (T, non-infected non-treated control plants (niCTR), infected non-treated control plants (iCTR), infected plants treated with methyl-jasmonate (iMeJA), salicylic acid (iSA) or benzo (1,2,3)-thiadiazole-7-carbothioic acid-S-methyl ester (iBTH)) on the concentration (μg.g^–1^) of micronutrients in leaf tissues. Significant *P* values (<0.05) are indicated in bold.

	Response variable	Factor	*F* ratio	*P* value
Micronutrients	B (μg.g^−1^ leaf)	Treatment (T)	2.96	**0.0396**
	Cu (μg.g^−1^ leaf)	Treatment (T)	5.77	**0.0020**
	Fe (μg.g^−1^ leaf)	Treatment (T)	6.10	**0.0014**
	Mn (μg.g^−1^ leaf)	Treatment (T)	4.74	**0.0055**
	Ni (μg.g^−1^ leaf)	Treatment (T)	7.86	**0.0003**
	Zn (μg.g^−1^ leaf)	Treatment (T)	6.35	**0.0011**

### Bacterial endophytic population

**Figure 7.**
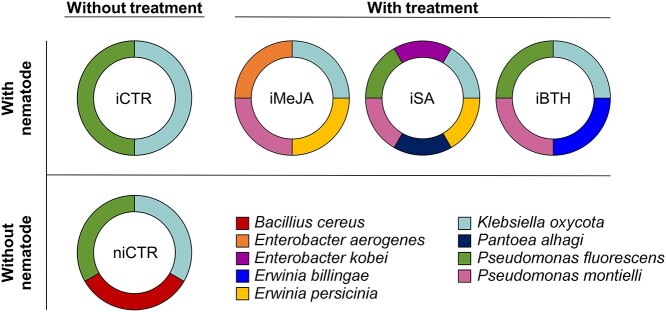
Bacterial populations in non-infected non-treated control plants (niCTR), infected non-treated control plants (iCTR), infected plants treated with methyl-jasmonate (iMeJA), salicylic acid (iSA) or benzo (1,2,3)-thiadiazole-7-carbothioic acid-S-methyl ester (iBTH) at the end of essay (at 35 dai).

The groups with the highest bacterial diversity were iSA, iMeJA and iBTH, which showed six different genera (corresponding to six (iSA) and four species (iMeJA and iBTH)) at the end of the experimental period (Figure 7). niCTR and iBTH presented three genera and iCTR presented two.

The main genera found were *Klebsiella*, represented by only one species (*Klebsiella oxycota*) present in all groups, and *Pseudomonas*, represented by two species, *Pseudomonas fluorescens* (present in niCTR, iCTR, iSA and iBTH) and *Pseudomonas monteilii* strain (only present in elicited plants). The second more abundant genus found was *Erwinia*, present in three of the five treatments, represented by two species, *Erwinia persicina* (in iMeJA and iSA) and *Erwinia billingiae* (iBTH). The third genus found was *Enterobacter*, represented by two species, *Enterobacter aerogenes* (iMeJA) and *Enterobacter kobei* (iSA). Finally, niCTR presented one specie from the genus *Bacillus* (*Bacillus cereus*), and iSA presented one species of the genus *Pantoea* (*Pantoea alhagi*).

## Discussion

### Plant elicitation, particularly with BTH, delays the development of the pine wilt disease

In the current work, a nematode population consisting of a mixture of adult and juvenile nematodes was used for plant inoculation. When pine plants are infected, two infection stages take place. In the first stage, most nematodes remain close to the site of inoculation and in the surrounding cortical tissues (Suzuki 1984). The second phase takes place only after there is a substantial reduction in oleoresin pressure and flow, and after transpiration rates are impaired; at this stage, there is an exponential increase in PWN population, and an increase in disease symptoms progression (Myers 1988, Suzuki 1984). In the current study, both the increase in the number of nematodes and the beginning of the appearance of foliar symptoms occurred at 21 dai, indicating the successful infection and multiplication of the PWN inside plant tissues over time. In a previous work, with 1-year-old (40–50 cm height) *P. pinaster* plants and maintained in a growth chamber (16 h light/8 h darkness photoperiod at 25/18°C), PWN infection progressed at higher rates between 7 and 14 dai (Nunes da Silva et al. 2021). This divergence could be a result of differences in plant age and size among the two experiments.

Plant elicitation decreased nematode density by up to 1.7-, 2.16- and 2.7-fold (for MeJA, SA and BTH, respectively) at the end of the experiments in comparison with non-elicited plants; BTH generally induced the lowest nematode density inside plant tissues at all time-points. Anthocyanins and carotenoids are involved in plant protection against photosystem damage (Elkhouni et al. 2018). It appears that the increase of these metabolites occurs at the beginning of the infection and at the end of the assay in iCTR plants as an attempt of the plants to minimize the adverse effects induced by the PWN. Regarding elicited plants, BTH led to the higher anthocyanin concentration, suggesting a higher antioxidant response to the PWN. In fact, infected plants treated with BTH presented lower MDA accumulation, indicating lower cellular damage, corroborated by the lower prevalence of foliar damage observed with this treatment throughout the study. The peroxidation of unsaturated lipids in cell membranes is caused by the necrotization of xylem parenchyma and cortex cells and to the destruction of phloem caused by PWN in susceptible species of the genus Pinus (Apel and Hirt 2004, Yamada 2008, Nunes da Silva et al. 2015). The accumulation of MDA, a secondary compound of lipid peroxidation reactions, is an indicator of cell damage induced by free radicals (Santos et al. 2012). Interestingly, despite preventing nematode reproduction in plants tissues, MeJA and SA elicitors did not prevent cellular damage, leading to increased MDA accumulation, corroborated by the highest foliar damage observed. This could be a result of the abrupt induction of plant defense mechanisms upon PWN infection (Agrawal et al. 2002). On the contrary, the infected plants treated with BTH presented a lower amount of accumulated MDA, indicating lower cellular damage, corroborated by the lower presence of foliar damage throughout our study. Polyphenols, together with anthocyanins and carotenoids are also secondary metabolites involved in plant tolerance to biotic stress (Kawaguchi 2006, Kuroda et al. 2011, Kusumoto et al. 2014, Gaspar et al. 2017). Total soluble phenols concentration gradually decreased until the end of the experimental period in iCTR and iSA, while increased progressively until the end of the trial in iMeJA; and iBTH. At 28 dai, a highly significant correlation was found between polyphenol concentration and greater colonization by PWN (Table 1), observing that there was both a greater number of nematodes and a greater accumulation of phenols in the iCTR than in the elicited plants (Figure 4A). This observation is consistent with a positive connection between migration of nematodes and the concentration of polyphenols, previously reported in a migration assay of PWN through wood tissues of 2-year-old branches from 10 years old plants (Zas et al. 2015).

### Minerals play an important role in tolerance against the PWN

It has been known that minerals and other nutrients have important specific functions in plant physiology, are essential for the metabolism of plants and play an important role in defending plants against biotic and abiotic stress. For this reason, it was chosen to measure mineral concentrations at 28 dai, coinciding with the point of greatest biotic stress indicated by the high accumulations of polyphenols and MDA (Figures 4A and 5), increased progression of disease symptoms and the increase more than double in the population of PWN (Figures 1 and 2). Many of these micronutrients are constituents of enzymes, specifically metalloproteins (Cu, Fe, Mn and Ni), others participate in the activation of enzymes (Mn and Zn) and some are constituents of plant cell walls and membranes (B and Zn) (Asher et al. 1991, Römheld and Marschner 1991, Bergmann and Caesar 1994, Welch and Shuman 1995, Mengel and Kirkby 2001, Kirkby and Römheld 2004, Epstein and Bloom 2005, Marschner 2011). It is known that the micronutrient composition of plant tissues in response to biotic and abiotic stresses is altered by application of elicitors (Ghassemi-Golezani and Farhangi-Abriz 2018, Débia et al. 2020). In the current work, there was a decrease in the concentration of B in infected plants (Figure 7A) in comparation with non-infected non-treated control plants (niCTR). So far, few works that have shown differences in the nutrient content in the tissues of pine plants infected with *B. xylophilus*, but Zou and Sun (2000) observed in Masson pine (*Pinus massoniana*) that there was a significant relationship between the tolerance to the nematode pest and mineral element content of the leaves and that this correlation changed at different stages of plant development. B is involved in carbohydrate metabolism, and when is in limited concentrations, the pentose phosphate pathway becomes predominant in carbohydrate degradation, leading to the formation of phenolic compounds (and tryptophan) by the shikimic acid pathway (Chen et al. 2014). The consequence of this is the accumulation of phenols and the increase in the activity of polyphenol oxidase, forming highly reactive intermediates, such as quinones (Ruíz et al. 1998, Ölçer and Kocaçalışkan 2007). These compounds, and also photo activated phenols, are highly effective in the production of superoxide radicals, which may damage membranes through lipid peroxidation. Therefore, the decrease in B caused by PWN infection seems to trigger oxidative stress, causing the accumulation of polyphenols and MDA. Contrastingly, Mn concentration increased (Figure 6D) in infected elicited plants, which also had the higher concentrations of phenols and increased lipid peroxidation (Table 1). Mn acts as a co-factor for several fundamental enzymes in the biosynthesis of secondary metabolites associated with the shikimic acid pathway, including phenolic aromatic amino acids, coumarins, lignin and flavonoids (Burnell 1988, Dordas 2008).

Cytochrome oxidase, catalase and peroxidase are Fe-dependent enzymes (Hänsch and Mendel 2009) and their activities often decrease under conditions of Fe deficiency (Mengel and Kirkby 2001). A drastic decrease in peroxidase activity with subsequent accumulation of phenolic substances has been reported under Fe limiting conditions (Römheld and Marschner 1981). Here, the lowest concentrations of Fe (Figure 6C) were observed in infected plants when compared to the non-infected control plants (niCTR), and the former were also the ones with the highest accumulation of phenols and lipid peroxidation (Table 1). This suggests that Fe plays an important role in the regulation of enzymes that could prevent the oxidation of tissues caused by PWN.

Cu is somewhat similar to Fe in that it forms highly stable chelates that allow the transfer of electrons. For this reason, it plays a role comparable to Fe in redox processes (Yruela 2005). Several Cu-containing proteins play critical roles in photosynthesis, respiration, superoxide radical detoxification and lignification (Droppa and Horváth 1990, Burkhead et al. 2009, Broadley et al. 2012). Cu deficiency causes the accumulation of phenols (Pilon et al. 2006, Hänsch and Mendel 2009); therefore, this mineral is important to increase plant tolerance to diseases (Schulten and Krämer 2017). In the current study, iCTR and iMeJA had the lowest Cu concentrations (Figure 6B). The moderate negative correlation between lipid peroxidation and foliar damage (Table 1) found in this trial supports that the lower Cu concentration may be related to the higher foliar damage (Figure 1C). Accordingly, iSA and iBTH presented higher concentrations of Cu (Figure 6B) and lower foliar damage (Figure 1C).

Here we observed an increase in lipid peroxidation (Figure 5), which may be related to higher concentrations of Ni (Figure 6E), as shown by the high correlation between these two parameters (Table 1). This relationship was also observed in *Solanum nigrum* and *Triticum durum* (Gajewska and Skłodowska 2007, Soares et al. 2016). Although mechanisms of detrimental impact of Ni on plants are not clearly understood, the phytotoxicity of this metal may be attributed to oxidative stress (Baccouch et al. 2001, Gonnelli et al. 2001).

Zn has an important role in plant metabolism, including effects on carbohydrate metabolism, protein synthesis, hormonal regulation and membrane integrity (Mengel and Kirkby 2001). Here we found a high correlation (Table 1) between higher Zn levels in non-treated infected control plants (iCTR) (Figure 6F), which also had higher concentrations of phenolic compounds (Figure 4A) and nematodes numbers (Figure 2). In *Kandelia obovata* a strong correlation was also found between Zn concentrations and the accumulation of phenolic compounds (Chen et al. 2019). On the other hand, it has also been suggested that plants grown in higher Zn levels have higher nematode levels, but this was probably due to negative effects on nematode antagonists (Georgieva et al. 2002). Therefore, it is possible that the increase in Zn is related to the higher number of PWNs isolated in the non-treated infected control plants (iCTR) as opposed to the lower concentration of this micronutrient and therefore a lower number of nematodes observed in the elicited plants.

### Elicitors induce changes in the bacterial community of Pinus pinaster

Treatment with elicitors usually leads to the activation of different plant defense pathways, such as SA-mediated SAR and JA-mediated ISR, two antagonistic defense-related pathways (Van Loon 2007, Bari and Jones 2009, Kolosova and Bohlmann 2012, Khan et al. 2015, Klessig et al. 2018, Tripathi et al. 2019). Once triggered, these pathways lead to the secretion of exudates that can alter the internal and external rhizosphere microbiome of plants (Lebeis et al. 2015, Liu et al. 2017, 2020, Mannaa et al. 2020). Given that the endophytic communities associated with PWD may play an important role in both the progression and suppression of the infection caused by the nematode (Alves et al. 2018, Kim et al. 2019), our assay aimed to study the modifications of bacterial communities after elicitor treatment.

We observed that the iCTR and the iBTH had two species in common (*Klebsiella oxytoca and P. fluorescens*), being the unique two species present in the iCTR. *K. oxytoca* is an endophytic plant growth-promoting strain (Hallmann et al. 1997); this nitrogen-fixing bacterium has been isolated from rice roots (Nguyen et al. 1989), and Roriz et al. (2011) suggested that it may be associated with the Portuguese region because previous works did not identify this bacteria in *Pinus pinaster*, and Alves et al. (2018) observed that there are changes in the bacterial community composition between the sampling sites. On another hand, *P. fluorescens*, that was present in all groups except in iMeJA; belongs to plant growth-promoting rhizobacteria (PGPR), which are associated to primary productivity through promotion of growth and triggering of induced systemic tolerance in plants (Hol et al. 2013, Panpatte et al. 2016). niCTR also presented *B. cereus,* previously described in Masson pine by having nematocidal activity (Li et al. 2020). *P. monteilii*, that has been placed in the *Pseudomonas putida* group (Anzai et al. 2000); was present in iMeJA, iSA and iBTH and it has been described that promotes plant growth, can induce systemic tolerance to root rot fungi and *P. putida* group can induce systemic resistance against PWN in seedlings and pine callus (Pandya and Desai 2014, Kim et al. 2019, Urooj et al. 2020). *E. billingiae* was only present in iBTH and is an epiphytic and saprophyte bacterium that may represent antagonists for biocontrol of fire blight (Kube et al. 2010), although it was also described as part of the rhizosphere of two species of the genus Pinus (*Pinus radiata* and *Pinus sylvestris*) (Nurmiaho-Lassila et al. 1997, Mesanza et al. 2019). Finally, the infected SA-elicited (iSA) and MeJA-elicited plants (iMeJA) were the ones with the greatest diversity of species, being *E. persicina* and *K. oxytoca*, the two common species in both treatments. *E. persicina* is a phytopathogenic bacteria that affects to common bean (*Phaseolus vulgaris*) but also other plant species (Zhang and Nan 2014). iMeJA presented *Enterobacter aerogenes,* an endophytic bacterium that colonizes plants and is suggested to improve plant growth (D’Alessandro et al. 2014). iSA presented *Enterobacter kobei*, a specie included in the *Enterobacter cloacae* complex (Humann et al. 2014), this complex is associated whit plant growth promotion (Khalifa et al. 2016, Macedo-Raygoza et al. 2019); and *Pantoea alhagi*, an endophytic bacterium with ability to improve plant growth and drought tolerance (Chen et al. 2017).

Although all these genera have been previously described as related to both pine and nematode (Proença et al. 2010, 2017, Roriz et al. 2011, Vicente et al. 2011), some species have been described for the first time associated with pine and the use of elicitors such as *B. cereus* (niCTR), *E. aerogenes* and *P. montielli* (iMeJA) and *E. kobei* and *P. alhagi* (iSA). The lower damage present in plants associated to the modulation of bacterial types and diversity due to elicitor application that we obtained in our assay was already shown in other studies in which different types of elicitors were applied in *Pinus densiflora* (Kim et al. 2019, Mannaa et al. 2020). Therefore, the application of elicitors increases and modifies the diversity of the *Pinus pinaster* microbiome favoring the proliferation of species that improve resistance against PWN, especially in plants treated with BTH, where the four species of bacteria isolated are associated with growth-promoting (*K. oxytoca* and *P. fluorescens*) and resistance to diseases such as that caused by the PWN (*E. billingiae* and *P. monteilii*).

## Conclusions

This study describes and compares changes occurring in *P*. *pinaster* plants infected with *B. xylophilus* after exogenous application of MeJA, SA and BTH. All elicited plants survived the inoculation assay, presenting fewer nematodes and foliar symptoms than control plants, thus decreasing the progression of PWD. Elicitation promoted defense mechanisms of pine plants against PWN, by increasing the concentrations of anthocyanins, carotenoids and phenolic compounds in plant tissues at specific stages following infection. However, high concentrations of MDA were observed in non-treated infected control plants and infected MeJA-elicited and SA-elicited plants, whereas in infected BTH-elicited plants the MDA concentrations were low, indicating lower cellular damage. Exogenous application of elicitors induced changes in the bacterial community promoting beneficial bacteria for the defense of *P. pinaster* against PWN, and altered the micronutrient concentrations like Mn that acts as a co-factor for several fundamental enzymes in the biosynthesis of secondary metabolites, and Zn whose lower concentration was correlated with the lower number of nematodes. This integrated study helps to elucidate the use of elicitors as a biocontrol method of the disease caused by the PWN and concludes that they may be beneficial for pines, for example in nurseries, because it could increase the production of plant defenses. However, our study indicates that other control methods should be used in conjunction with elicitors for a proper management of this disease in a forest environment.

## Supplementary Material

Supplemental_data_for_online_publication_Figure_S1_tpac088

Supplemental_data_for_online_publication_Figure_S1_tpac088
