# Supplementary material for: Evaluation of plant elicitation with methyl-jasmonate, salicylic acid and benzo (1,2,3)-thiadiazole-7-carbothioic acid-S-methyl ester for the sustainable management of the pine wilt disease
Source: Tree Physiol. 2022 Jul 22;42(12):2596–613. doi: 10.1093/treephys/tpac088 (PMC11648887; doi:10.1093/treephys/tpac088)
Supplement: Supplemental_data_for_online_publication_Figure_S1_tpac088 [file supplemental_data_for_online_publication_figure_s1_tpac088.docx]

**Supplemental data for online publication**

**Figure 1S (A)** Chlorophyll-A (µmol. g-1 leaf) and **(B)** Chlorophyll-B (µmol. g-1 leaf) in infected non-treated control trees (iCTR), infected trees treated with methyl-jasmonate (iMeJA), salicylic acid (iSA) or benzo (1,2,3)-thiadiazole-7-carbothioic acid-S-methyl ester (iBTH) (7, 14, 21, 28 and 35 days after inoculation). Values represent the mean of 4 biological replicates ± standard error of the mean. Significance levels of treatments and time-point for malondialdehyde: ***, P < 0.001; **, P < 0.01; *, P < 0.05; ns, not significant.
