# Supplementary figures and images for: Evaluation of plant elicitation with methyl-jasmonate, salicylic acid and benzo (1,2,3)-thiadiazole-7-carbothioic acid-S-methyl ester for the sustainable management of the pine wilt disease
Source: Tree Physiol. 2022 Jul 22;42(12):2596–613. doi: 10.1093/treephys/tpac088 (PMC11648887; doi:10.1093/treephys/tpac088)

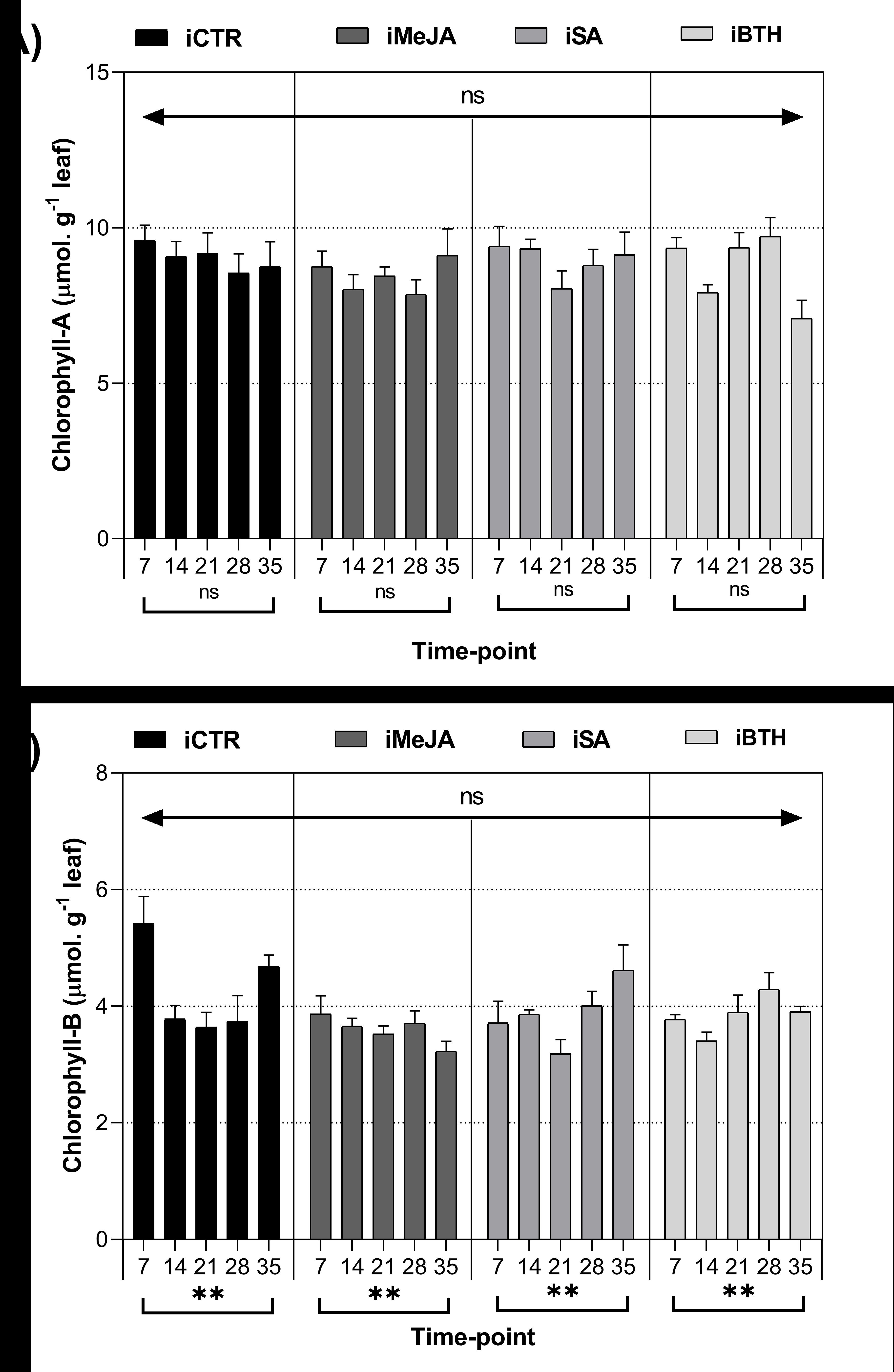

Supplement: Supplemental_data_for_online_publication_Figure_S1_tpac088 [file supplemental_data_for_online_publication_figure_s1_tpac088.jpeg]
